# Adenoid cystic carcinoma in an 86-year-old male patient: A rare case report

**DOI:** 10.1016/j.radcr.2025.05.066

**Published:** 2025-06-21

**Authors:** Rim Alami, Reyzane El Mjabber, Malak Chahid, Meryem Naciri, Zineb Dahbi, Fadila Kouhen, Sanaa El Majjaoui, Nabil Ismaili, Asmaa Naim

**Affiliations:** a Mohammed VI Faculty of Medicine, Mohammed VI University of Health Sciences, Ave Mohamed Taieb Naciri, Casablanca, 82403, Morocco; b Department of radiotherapy, International University Hospital Cheikh Khalifa, Casablanca, Morocco; c Mohammed VI Faculty of Medicine, Mohammed VI University of Sciences and Health (UM6SS), Rabat, Morocco; d Laboratory of Neurooncology, Oncogenetic and Personalized Medicine, Faculty of Medicine, Mohammed VI University of Sciences and Health (UM6SS), Casablanca, Morocco; e Department of medical oncology, International University Hospital Cheikh Khalifa, Casablanca, Morocco; f Simulation Research Laboratory in Sciences and Health, Mohammed VI University of Health Sciences (UM6SS), Casablanca, Morocco

**Keywords:** Adenoid cystic carcinoma, Salivary gland tumors, Radiation therapy, Head and neck cancers, Case report

## Abstract

Adenoid cystic carcinoma (ACC) is characterized by slow growth, a marked tendency for local invasion, and a high rate of recurrence. In this context, we present a noteworthy case of ACC in an 86-year-old North African male, arising in the retromolar trigone-an exceptionally rare location. This case report outlines the clinical presentation, diagnostic workup, therapeutic interventions, and patient outcomes associated with ACC, highlighting the complexities inherent in the management of this challenging malignancy. Our findings emphasize the critical role of multidisciplinary collaboration, ongoing research, and individualized treatment strategies to optimize ACC management and enhance patient care.

## Introduction

Adenoid cystic carcinoma (ACC) presents a distinct and significant challenge in oncology due to its indolent yet aggressive nature, marked by local invasion and a high rate of recurrence. Although ACC predominantly arises in the major salivary glands, it can also manifest in various anatomical locations, complicating both diagnosis and treatment. We report an intriguing case of ACC located in the retromolar trigone—an exceedingly rare site—in an 86-year-old North African male patient. This case study aims to elucidate the clinical presentation, diagnostic approach, therapeutic strategies, and outcomes associated with ACC, emphasizing the complexities of managing this rare malignancy. Our discussion incorporates findings from recent studies, highlighting the importance of a multidisciplinary approach and the ongoing need for research to optimize treatment strategies for ACC patients.

## Patient information

An 86-year-old North African male patient, with no significant comorbidities, presented with a solid mass in the retromolar trigone that had progressively enlarged over 2 years and had previously been neglected. The lesion was accompanied by unquantified weight loss, attributed to partial obstruction of the digestive tract and masticatory difficulties. Three months prior to consultation, the patient developed a nasal speech pattern, and the tumor began to bleed spontaneously.

## Clinical findings

**Fig. 1:**
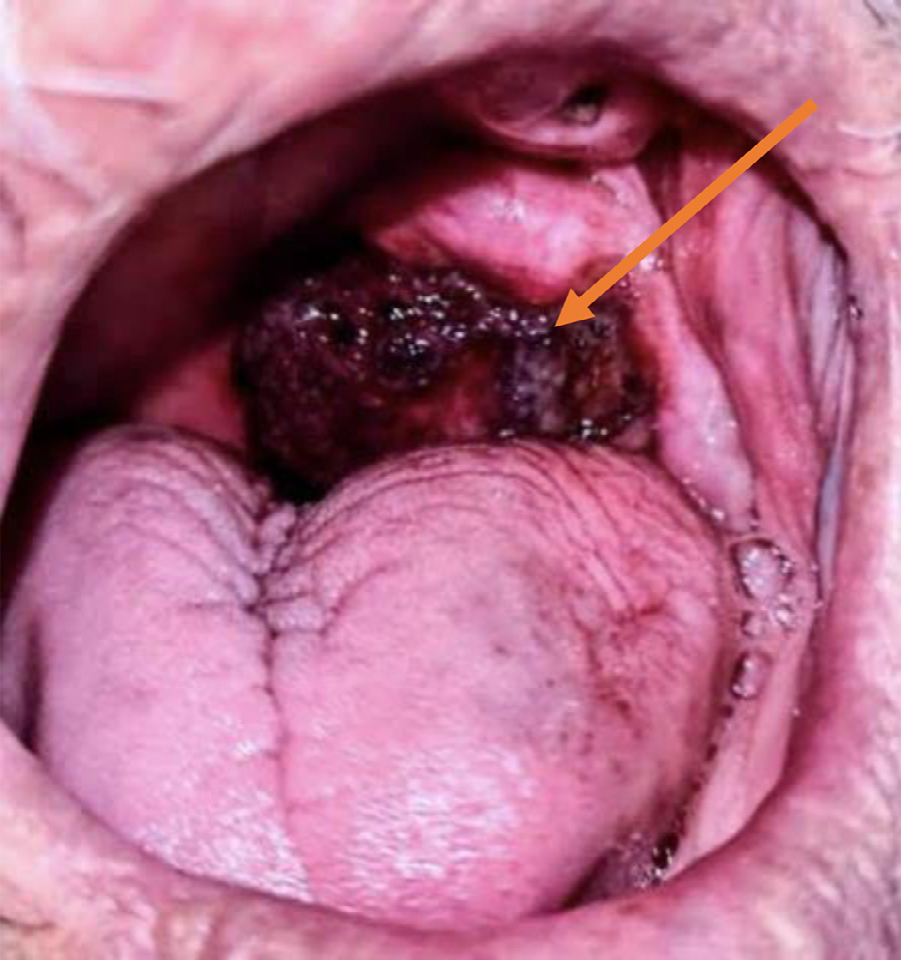
Clinical endobuccal view of the exophytic lesion of the left retromolar trigone (orange arrow).

Clinically, the patient was in good general health, with a performance status (PS) score of 0. Intraoral examination revealed a painful, dark-red exophytic lesion with a rough, heterogeneous surface located on the left buccal mucosa, measuring approximately 3 × 2 cm. The lesion was firm, bled upon contact, and emitted an unpleasant odor (Fig. 1). Cervical examination showed no lymphadenopathy, and the remainder of the physical assessment was unremarkable, with no signs of distant metastasis.

## Diagnostic assessment

An incisional endobuccal biopsy was performed under local anesthesia. Histopathological examination of the specimen revealed a malignant neoplastic proliferation of stratified squamous epithelial cells, arranged in sheets or islands and infiltrating the connective tissue with a cribriform pattern, prompting further immunohistochemical evaluation. Immunohistochemistry demonstrated positive staining for anti-CK7, anti-P63, and anti-smooth muscle actin antibodies, with a Ki-67 proliferation index below 5%. Focal positivity was observed for anti-CD117, while anti-DOG-1 was negative. The final diagnosis was adenoid cystic carcinoma.

**Fig. 2:**
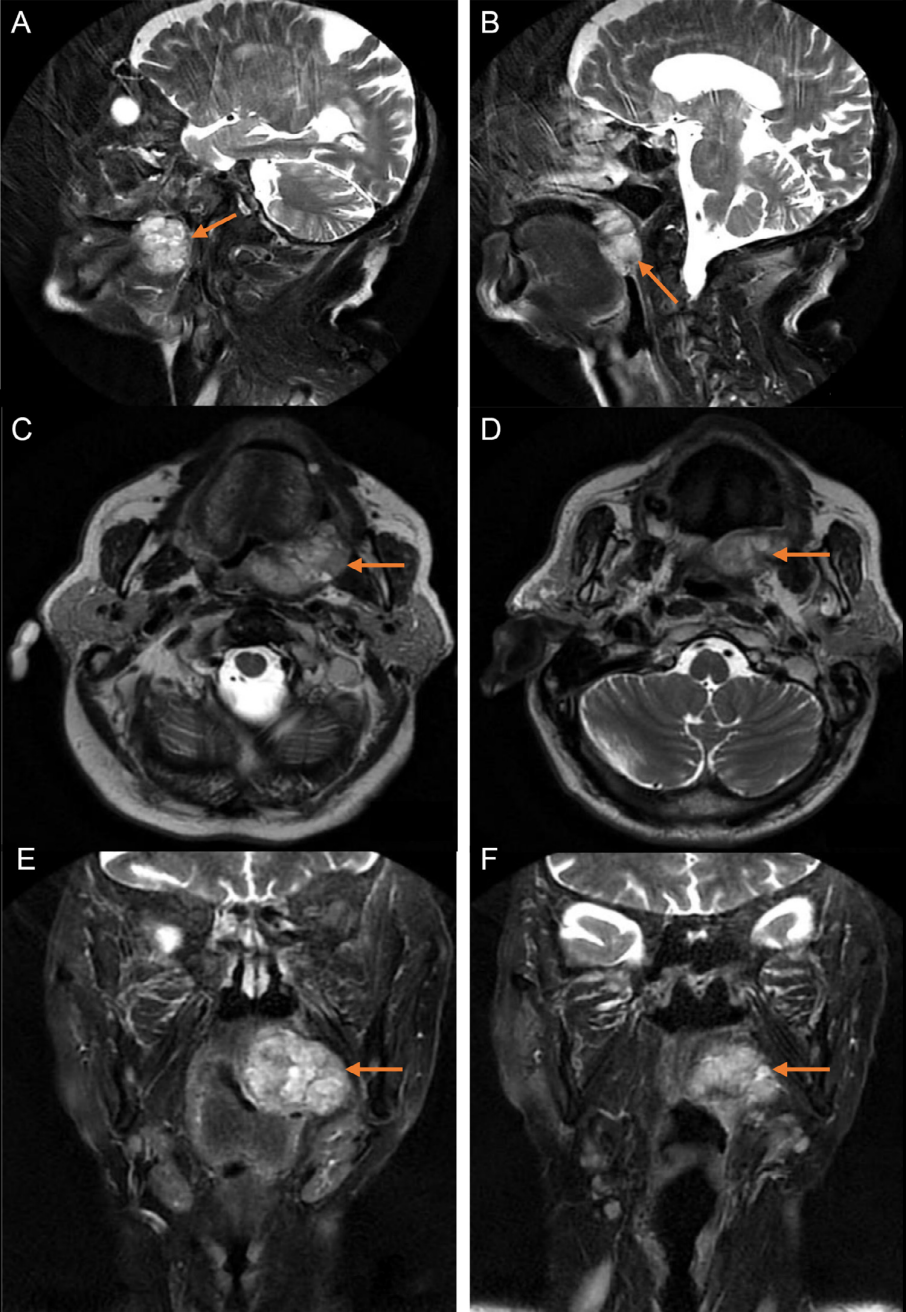
Initial head and neck MRI of our patient revealing a tumor of 39.4 × 21.4 × 39.7 mm (orange arrows). (A and B) Sagittal T2 propeller sequence. (C and D) Axial T2 propeller sequence. (E and F) Coronal T2 propeller sequence.

The patient subsequently underwent cervicofacial MRI (Fig. 2), which demonstrated:

• A contrast-enhancing mass in the left retromolar trigone, exhibiting T2 hyperintensity, intermediate signal on Diffusion-Weighted Imaging (DWI), isointensity on T1, and heterogeneous enhancement postgadolinium administration, measuring 39 × 39 × 33 mm.
• Superior extension into the left bony palate.
• Anterior intimate contact with the fixed base of the left tongue, displacing it anteriorly.
• Medial narrowing of the oropharyngeal lumen.
• Lateral intimate contact with the palatoglossus and pterygoid muscles, as well as the horizontal ramus of the mandible.
• Posterior invasion of the tonsillar region and parapharyngeal space.
• Mass effect on the nasopharynx and esophagus.
• No evidence of perineural invasion (PNI), lymphadenopathy, or paravertebral soft tissue abnormalities.

The initial assessment included a thoracic CT scan, which revealed no evidence of distant metastasis.

The patient was staged as T4N0M0 according to the eighth edition of the AJCC Cancer Staging Manual. Following multidisciplinary discussion by the otorhinolaryngology board, exclusive radiotherapy was selected as the treatment approach due to the locally advanced nature of the tumor.

## Therapeutic intervention

The patient underwent external beam radiotherapy utilizing Volumetric Modulated Arc Therapy (VMAT). Two planning target volumes (PTVs) were defined: PTV50, encompassing the tumor, its margins, and the full nerve pathways of V2, V3, and IX up to their respective skull base foramina; and PTV70, including the gross tumor volume with margins. Standard fractionated radiotherapy was administered at a dose of 2 Gy per fraction, 5 fractions per week.

During radiotherapy, the patient developed Grade II oral mucositis and Grade II radiodermatitis, as classified by the Common Terminology Criteria for Adverse Events (CTCAE) version 5.0. These acute toxicities led to minor weight loss but did not necessitate interruption of treatment.

## Follow-up and outcomes

Three months after completion of radiotherapy, clinical and radiological evaluation demonstrated partial remission. MRI findings included:

• Reduction in the size of the left retromolar trigone mass, with decreased T2 and DWI hyperintensity, T1 isointensity, peripheral heterogeneous enhancement postgadolinium, and a new measurement of 24 × 15 × 19 mm compared to 39 × 38 × 21 mm previously.
• Resolution of superior invasion into the left bony palate.
• Persistent but nondisplacing anterior contact with the fixed base of the left tongue.
• Improvement in oropharyngeal lumen narrowing.
• Decreased external contact with the palatoglossus and pterygoid muscles, and loss of contact with the horizontal ramus of the mandible.
• Reduction in posterior invasion of the tonsillar region and parapharyngeal space.
• Resolution of mass effect on the nasopharynx and esophagus.

**Fig. 3:**
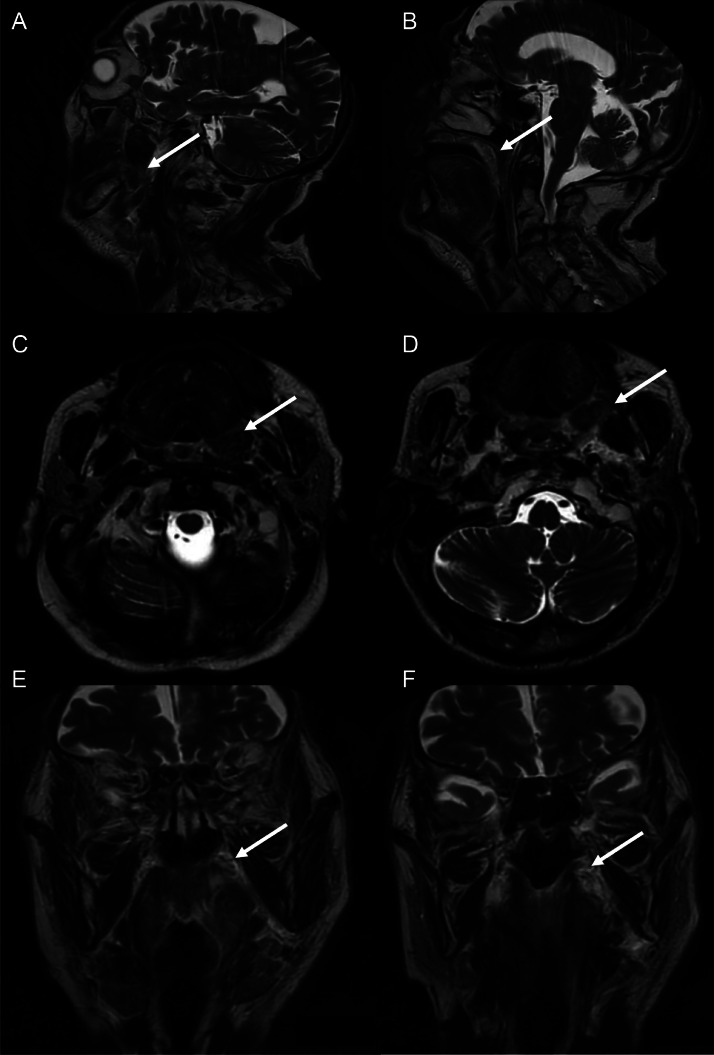
Head and neck MRI of our patient showing partial remission (white arrows). (A and B) Sagittal T2 propeller sequence. (C and D) Axial T2 propeller sequence. (E and F) Coronal T2 propeller sequence.

The patient exhibited persistent late adverse effects, including cutaneous fibrosis and mild trismus. Fig. 3

## Discussion

Adenoid cystic carcinoma (ACC) primarily arises in the major or minor salivary glands, accounting for approximately 22% of all salivary gland malignancies. However, it may also originate from other anatomical sites containing glandular structures, such as the cervix, breast, colon, and prostate [[1], [2], [3]]. The occurrence of ACC in accessory salivary glands is uncommon, with involvement of the retromolar trigone being particularly rare, representing only 6.4% of cases [[4], [5]].

Risk factors for ACC include advanced age and specific genetic mutations, which may serve as potential therapeutic targets [6]. Meta-analytical research by Li Q. and colleagues has established a correlation between p53 gene expression and survival rates in patients with adenoid cystic carcinoma of the salivary glands [7].

Histologically, ACC is characterized by cystic and glandular growth patterns and is typically classified as a low-grade malignancy. Nonetheless, it can demonstrate aggressive behavior, infiltrating adjacent lymph nodes and perineural spaces-the sheaths surrounding nerve fibers. Histopathological examination frequently reveals perineural invasion (PNI), present in approximately 50% of cases, underscoring its high metastatic potential. The lungs are the most common site of distant metastasis [[3], [8], [9]].

PNI is sometimes associated with “skip” lesions along the nerve, significantly increasing the risk of recurrence after resection, even when negative margins are achieved [10]. Due to its indolent progression, ACC often presents at an advanced local or metastatic stage, complicating surgical management and predisposing to frequent late recurrences [3].

MRI is the modality of choice for evaluating soft tissue and perineural tumor extension. Multiparametric sequences, including DWI and Dynamic Contrast-Enhanced (DCE) MRI, are particularly valuable. MRI is instrumental in detecting and delineating the extent of PNI, which may appear as asymmetric nerve enlargement or enhancement, obliteration of perineural fat planes, or destruction or widening of neural foramina [11].

Radiological evidence of PNI in asymptomatic patients is rare. Differentiating physiological enhancement of the perineural venous plexus from true PNI is essential, as conditions such as meningiomas, schwannomas, and granulomatous diseases affecting the skull base foramina can mimic PNI [[11], [12]].

Histopathologically, cribriform areas are commonly observed in both ACC and polymorphous low-grade adenocarcinoma, complicating their distinction. However, immunohistochemistry can assist in differentiating between these entities [[13], [14]].

Given the rarity of ACC, patient cases should be reviewed by a specialized multidisciplinary board to determine the optimal treatment strategy. Surgical resection remains the preferred treatment option when feasible. Indications for postoperative radiotherapy include incomplete excision, positive or close margins, and PNI; some centers advocate for its routine use postoperatively [3]. In the present case, due to the tumor’s location and size, surgical intervention was not considered.

Radiotherapy is the primary therapeutic modality for unresectable, locally advanced tumors, as illustrated in our case. Notably, ACC’s propensity for neural spread necessitates the inclusion of involved nerves and their origins at the skull base in the radiotherapy planning volume, as well as additional cranial nerves potentially at risk [15].

Given the relatively low probability of regional lymph node metastasis [16], inclusion in the treatment volume is reserved for advanced T-stages or early T stages with histologically confirmed or imaging highly suspected lymph node disease [[15], [17], [18]].

Palliative chemotherapy is considered for adenoid cystic carcinoma when surgery or radiotherapy are not feasible, but its effectiveness remains limited, with low objective response rates in both locally advanced and metastatic cases [[6], [19]]. For patients with oligometastatic disease, options such as metastasectomy or Stereotactic Body Radiotherapy (SBRT) are available; the ongoing SOLAR phase II trial is investigating the impact of SBRT on disease progression, quality of life, and overall survival in ACC patients with 1 to 5 metastases, with results expected in 2028 [20]Tumor molecular profiling is increasingly important for selecting systemic therapies, as targeted treatments may be effective when specific molecular abnormalities, such as alterations in the MDM2 signaling pathway, are identified [21]. Prognosis depends on tumor size, location, and stage at diagnosis, and regular follow-up is essential due to the high recurrence rate, which ranges from 30% to 75% at 5 and 10 years [22].

## Conclusion

This case concerns an elderly male patient with adenoid cystic carcinoma (ACC) of the retromolar trigone. Although ACC most commonly arises in major salivary glands, it can develop in various anatomical sites, requiring a comprehensive diagnostic approach. Management is challenging due to its indolent growth, high recurrence rate, and metastatic potential. In this case, radiotherapy using VMAT yielded favorable outcomes, demonstrating its efficacy in treating unresectable or advanced ACC. Our findings highlight the importance of multidisciplinary collaboration and ongoing research to improve therapeutic strategies and patient outcomes.

Advances in tumor molecular profiling and targeted therapies hold promise for personalized treatment regimens. Given ACC’s tendency for recurrence, long-term surveillance with regular follow-up is essential. This case adds to the growing literature on ACC management, emphasizing thorough evaluation and individualized care planning.

## Patient consent

Written informed consent was obtained from the patient for publication of this case report and any accompanying images. A copy of the written consent is available for review by the Editor-in-Chief of this journal.
